# Advancing Antibiotic Residue Analysis: LC-MS/MS Methodology for Ticarcillin Degradation Products in Tomato Leaves

**DOI:** 10.3390/antibiotics13020133

**Published:** 2024-01-29

**Authors:** Muhammad K. Hakeem, Sampathkumar Elangovan, Mohammed Rafi, Suja George, Iltaf Shah, Khaled M. A. Amiri

**Affiliations:** 1 Department of Chemistry, College of Science, United Arab Emirates University (UAEU), Al-Ain P.O. Box 15551, United Arab Emirates; 700039966@uaeu.ac.ae (M.K.H.); sampath@uaeu.ac.ae (S.E.); 2 Khalifa Center for Genetic Engineering and Biotechnology, United Arab Emirates University, Al-Ain P.O. Box 15551, United Arab Emirates; rafi.m@uaeu.ac.ae (M.R.); sujageorge@uaeu.ac.ae (S.G.); 3 Department of Biology, College of Science, United Arab Emirates University (UAEU), Al-Ain P.O. Box 15551, United Arab Emirates

**Keywords:** LC-MS/MS, ticarcillin, thiophene-2-acetic acid, thiophene-3-acetic acid, antibiotic residue analysis

## Abstract

The indiscriminate use of antibiotics in agriculture has raised concerns about antibiotic residues in food products, necessitating robust analytical methods for detection and quantification. In this study, our primary aim was to develop a robust and advanced liquid chromatography-tandem mass spectrometry (LC-MS/MS) methodology specifically designed for the accurate quantification of ticarcillin degradation products in tomato leaves. The choice of ticarcillin as the target analyte stems from its frequent use in agriculture and the potential formation of degradation products, which can pose a threat to food safety. The use of tomatoes as the target sample matrix in this study is justified by their significance in human diets, their widespread cultivation, and their suitability as a model for assessing antibiotic residue dynamics in diverse agricultural environments. By optimizing the MS/MS parameters, the study successfully demonstrates the practicality and reliability of the employed LC-MS/MS method in accurately assessing ticarcillin degradation product (Thiophene-2-Acetic acid and Thiophene-3-Acetic acid) levels. The chromatographic separation was achieved using a specialized column, ensuring high resolution and sensitivity in detecting analytes. Multiple reaction monitoring (MRM) data acquisition was employed to enhance the selectivity and accuracy of the analysis. The developed method exhibited excellent linearity and precision, meeting the stringent requirements for antibiotic residue analysis in complex matrices. Key outcomes of this study include the successful identification and quantification of ticarcillin and its degradation products in tomato leaves, providing crucial insights into the fate of this antibiotic in agricultural settings. The methodology’s applicability was further demonstrated by analyzing real-world samples, highlighting its potential for routine monitoring and ensuring food safety compliance. In summary, our study constitutes a noteworthy advancement in the domain of antibiotic residue analysis, offering a reliable method for quantifying ticarcillin degradation products in tomato leaves. The optimized parameters and MRM-based LC-MS/MS approach enhance the precision and sensitivity of the analysis, opening up opportunities for further studies in the assessment of antibiotic residues in agricultural ecosystems.

## 1. Introduction

In the world of agriculture, the quest for innovative solutions to enhance crop yield, quality, and sustainability is ongoing. One promising avenue that has gained significant attention is the use of plant growth regulators (PGRs) [1,2]. These natural or synthetic compounds, also known as plant hormones or phytohormones, have the ability to influence various aspects of the development and growth of plants [3]. From seed germination to flowering and fruiting, PGRs play a pivotal role in regulating these processes [4]. Plant growth regulators can be classified into auxins, cytokinins, gibberellins, ethylene, and abscisic acid. Auxins, for example, are responsible for promoting cell division and elongation, while gibberellins play a key role in regulating plant height and flowering [5]. Cytokinins, on the other hand, are involved in the control of cell division and differentiation [6]. Each of these plant hormones contributes to the overall growth and development of plants.

In recent times, there has been a notable surge in demand for plant growth regulators, which can be attributed to several different factors. One key driver is the increasing demand for higher agricultural productivity to feed the growing global population. PGRs offer a promising solution by enhancing crop growth, improving stress tolerance, and increasing overall productivity [7]. Another factor fueling the demand for PGRs is that they provide an alternative approach by reducing the need for excessive chemical inputs while still achieving desired crop outcomes [8]. By promoting healthy plant growth and enhancing natural defenses, PGRs enable farmers to adopt more environmentally friendly farming practices.

As research and technology advancements continue to unfold, the potential applications and benefits of PGRs are expanding. The development of innovative and eco-friendly PGR formulations is a key focus, aligning with the growing demand for sustainable agriculture [9]. Moreover, gene editing techniques allow for precise modifications in plant physiology, enabling the incorporation of gene-edited PGRs into plants [10]. By tailoring PGRs to specific crops and growth conditions, farmers can optimize their agricultural output and address specific challenges [11]. Plant growth regulators are powerful tools, offering the potential to enhance crop yield, quality, and sustainability. In this regard, auxins have been widely recognized to be significant regulators of numerous events in plant development and growth. Auxins play a significant role in controlling various biological activities in plants, including elongation, differentiation, and cell division [12,13,14,15]. Auxins also play a significant role in the development of adventitious roots [16,17] and the regulation of apical dominance [18,19]. Furthermore by manipulating auxin levels, it is possible to control flowering time and enhance fruit production [20].

In nature, the most well-known occurring auxin is indole-3-acetic acid (IAA). It regulates multiple biological functions, such as differentiation, cell division, and elongation [21]. Phenylacetic acid (PAA), 4-chloroindole-3-acetic acid (4-Cl-IAA), and indole-3-butyric acid (IBA) are some other naturally occurring auxins. In plant tissue culture, synthetic auxins such as picloram and 2,4-Dichlorophenoxyacetic acid (2,4-D) as well as naphthaleneacetic acid (NAA) are frequently employed as plant growth regulators [22].

The knowledge of auxin’s role in plant growth and development has practical applications in agriculture. By understanding the mechanisms of auxin action, it is possible to develop strategies to enhance crop production and quality. In agrobacterium-mediated plant transformation research, the antibiotic timentin is frequently employed to inhibit the growth of *Agrobacterium tumefaciens* following co-cultivation [23]. Timentin is a formulation that provides stability to the β-lactam ring in the broad-spectrum semi-synthetic β-lactam antibiotic ticarcillin by combining it with clavulanic acid, a β-Lactamase competitive inhibitor [24,25]. Timentin has been demonstrated to have a favorable impact on shoot regeneration in several plant species, including London plane trees, tomato, and tobacco, along with a few other β-lactam ring-containing antibiotics like penicillin G and carbenicillin [23,26,27,28]. Conversely, other research has also shown that these antibiotics have no appreciable impact on the regeneration of shoots in species like tobacco and Siberian elm [29,30].

**Figure 1:**
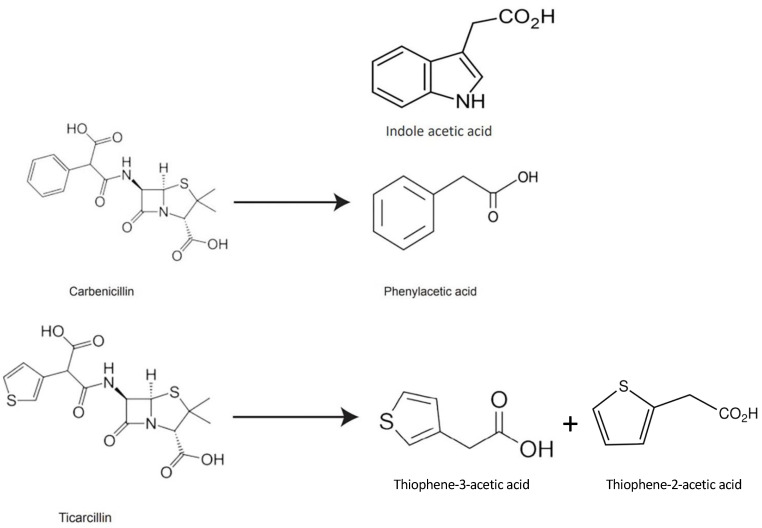
Chemical structures of carbenicillin, phenylacetic acid, indole acetic acid, ticarcillin, and thiophene acetic acid (T2AA, T3AA) [24].

The structural characteristics of β-lactam antibiotics are comparable to those of auxins, and the products of their decomposition may behave similarly to auxins in the culture media [31,32]. In our previous study, we postulated that Thiophene-3-Acetic acid (T3AA) may be a possible breakdown product of ticarcillin, based on the structure of ticarcillin (Figure 1 [24]). TAA, an organosulfur chemical, has two isomers: Thiophene-2-Acetic acid (T2AA) and Thiophene-3-Acetic acid (T3AA). Its molecular formula is C_6_H_6_O_2_S.

Tomato (*Solanum lycopersicum*) is an economically important crop, with extensive applications in the food industry. Organogenesis, the formation of new organs from plant tissues in culture, plays a vital role in tomato propagation and genetic transformation. The regulation of organogenesis through the application of exogenous substances, such as auxin analogs, can have a profound impact on tomato in vitro regeneration [33,34]. Our prior research has shown that the presence of timentin increases the rate of shoot regeneration in tomato cotyledon explants [24]. To verify the degradation of timentin into Thiophene-2-Acetic acid and Thiophene-3-Acetic acid, we regenerated tomato cotyledon explants in culture media designed for shoot regeneration in the presence and absence of timentin. We then measured the presence of Thiophene-2-Acetic acid and Thiophene-3-Acetic acid in the samples.

As far as we are aware, there is currently no reported LC-MS/MS methodology available for detecting ticarcillin and its metabolites in tomato leaves. Given that TAA has been recently demonstrated as an auxin analog capable of regulating organogenesis in tomato [24], a standardized method for the detection and quantification of TAA isomers in plant tissues is essential in deciphering mechanisms of organogenesis mediated by TAA. In addition, standardization of such a protocol would enable the detection of ticarcillin degradation products in other plant species. This analytical approach can also be instrumental in assessing antibiotic residue levels in agricultural products and ensuring food safety and security.

## 2. Results and Discussion

### 2.1. Validation Results

**Figure 2:**
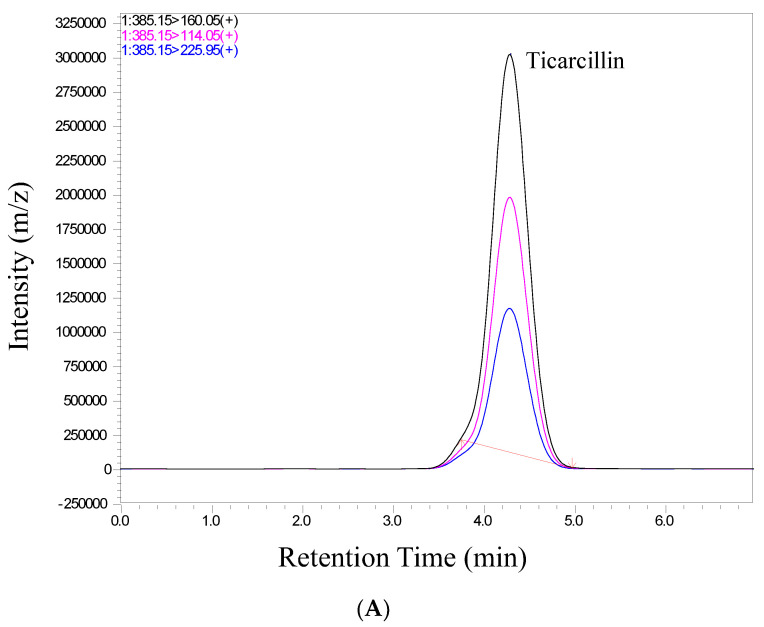

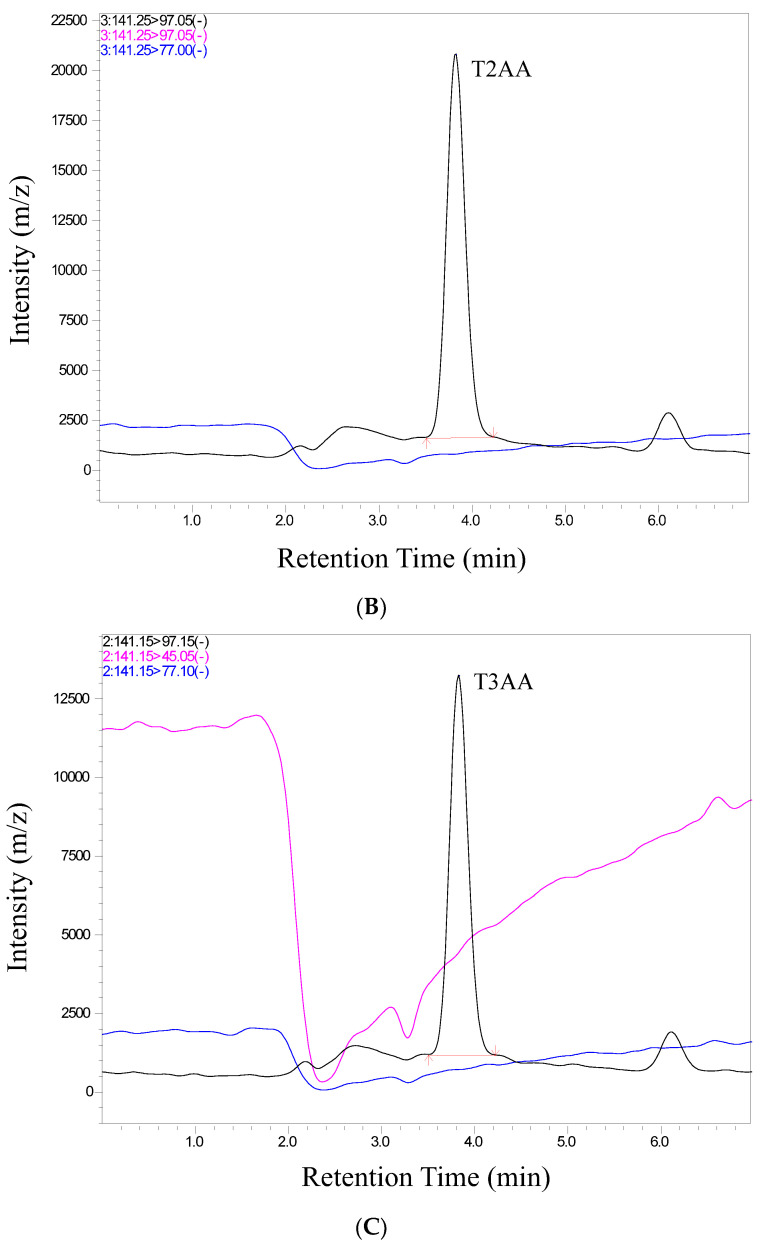
Representative chromatograms of (**A**) ticarcillin (**B**) Thiophene–2–Acetic acid, and (**C**) Thiophene–3–Acetic acid from a real sample analyzed on 14th day.

We conducted a thorough validation of the analytical method employed in this study to quantify ticarcillin and its metabolites. The validation process included an assessment of retention time and relative abundance (intensity), ensuring the robustness and reliability of our analytical approach. Figure 2 provides a visual representation of individual chromatograms, illustrating the distinct peaks corresponding to ticarcillin, Thiophene-2-Acetic acid, and Thiophene-3-Acetic acid. These peaks align precisely with their specific retention times for all analytes, showcasing the method’s ability to separate and identify target compounds efficiently. Notably, the absence of interference from matrix peaks in the chromatograms ensures the specificity of our method. This is crucial for accurate quantification, as it minimizes the likelihood of false positives or distorted results due to the presence of unwanted compounds. Moreover, the well-defined and resolved peaks in Figure 2 not only demonstrate the method’s selectivity but also highlight its robustness in differentiating the analytes of interest from potential interferences. This selectivity is pivotal for ensuring the accuracy and reliability of the quantitative results in complex sample matrices.

In scrutinizing the chromatographic data for ticarcillin degradation products, a comprehensive analysis was performed to discern potential interferences or co-elution at specific retention times. The chromatographic conditions employed in this study not only facilitated the effective separation, identification, and quantitation of ticarcillin and its metabolites but also provided valuable insights into the discriminating capabilities of the LC-MS/MS methodology. Through meticulous chromatographic separation, noticeable distinctions in the retention times of analytes were observed. Figure 2 serves as a visual representation of the observed differences, enabling the identification of individual analytes based on their elution times and abundance (area) (see Table S2). This distinctive elution behavior aids in the accurate characterization and quantification of the targeted substances. The methodology’s efficacy in discerning subtle variations in retention times enhances the precision of the analysis, contributing to a more robust understanding of ticarcillin degradation products.

### 2.2. Calibration Curve

Accurate quantitation is fundamental to the reliability of analytical results, and our approach to TAA isomer analysis centers on the establishment of a robust calibration curve. The foundation of this calibration curve lies in a meticulously prepared high-concentration stock solution of TAA, boasting a concentration of 1 mg/mL. This stock solution forms the basis for generating a comprehensive range of calibration standards, strategically spanning from 500.0 ng/mL to a maximum of 3500.0 ng/mL. The accuracy of our calibration standards is of utmost importance, and to achieve this, a carefully designed dilution process is employed. We utilize a specialized diluent solution composed of a precise 90:10 ratio of Milli-Q water to acetonitrile, fortified with 0.1% formic acid. This specific diluent composition serves as a critical factor in achieving not only accurate quantitation but also in addressing potential matrix effects and elevating the overall precision of our analytical approach. Furthermore, the selection of this diluent is not arbitrary; rather, it is grounded in a keen understanding of the intricate dynamics involved in TAA isomer analysis. The 90:10 ratio of Milli-Q water to acetonitrile provides an optimal balance between solubility and sensitivity, while the inclusion of 0.1% formic acid contributes to the mitigation of matrix effects. Formic acid, being a volatile acid, facilitates ionization and enhances the overall performance of the mass spectrometric analysis. The careful preparation and selection of the diluent underscore our commitment to minimizing potential sources of error and ensuring the reproducibility of our results. This methodological precision not only supports the accuracy of our calibration standards but also encourages the robustness of the entire analytical process. As a result, the calibration curve becomes a reliable tool for quantifying TAA isomers across a dynamic concentration range, providing a solid foundation for the subsequent analysis and interpretation of experimental results. This meticulous calibration approach strengthens the validity of our findings and contributes to the overall quality and integrity of our study.

Our results revealed a remarkably robust linear range spanning from 500.0 to 3500.0 ng/mL, underscoring the accuracy and precision of the developed method (see Figure S1). The correlation coefficient (R^2^ = 0.99) further attests to the strength of the linear relationship between the analyte concentrations and their respective response intensities. This high coefficient of determination emphasizes the method’s suitability for accurately quantifying ticarcillin and its metabolites across a wide concentration range.

### 2.3. Sample Analysis

**Figure 3:**
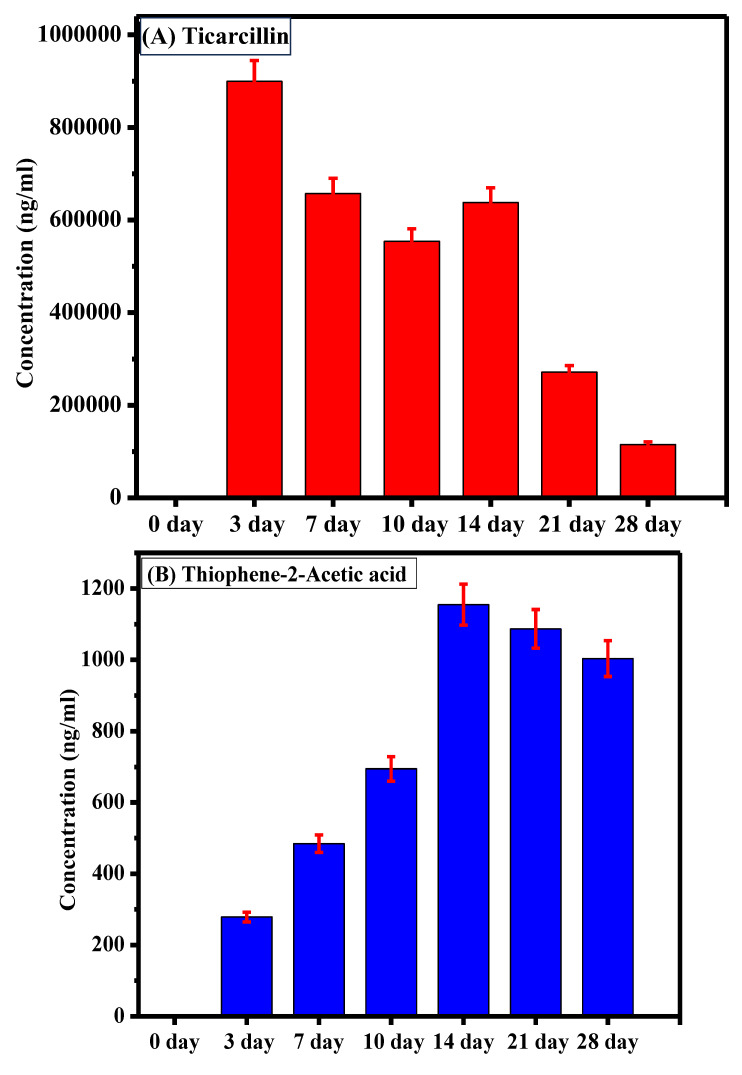

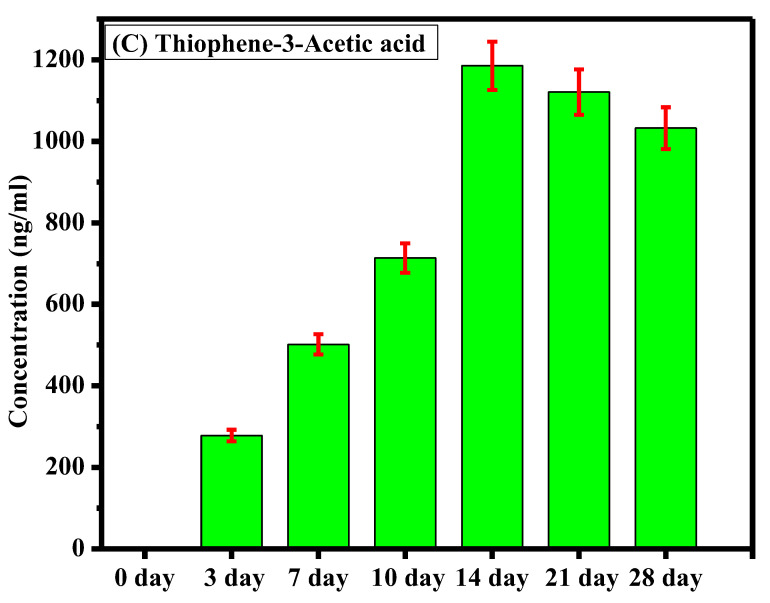
Comparative representation of concentration of (**A**) ticarcillin (**B**) Thiophene–2–Acetic acid, and (**C**) Thiophene–3–Acetic acid on different days.

The sample analysis aimed to assess the degradation of ticarcillin into its primary metabolites (T2AA and T3AA). Initially, at the onset of the experiment, no detectable concentrations of any analyte were observed, indicating the pristine state of the ticarcillin sample. As the experiment progressed, by the third day, the emergence of ticarcillin metabolites became apparent, suggesting the initiation of the degradation process. This observation was further substantiated by the increasing concentrations of T2AA and T3AA over the experimental period. The culmination of this degradation process was most notable on the 14th day, revealing the highest concentration of ticarcillin metabolites, as graphically depicted in Figure 3. The data clearly illustrate a progressive increase in the concentrations of T2AA and T3AA, reaching a peak on the 14th day. This finding suggests that the maximum degradation of ticarcillin occurs within this timeframe.

The observed time-dependent increase in concentration of ticarcillin metabolites implies a temporal pattern in the degradation process, with the 14th day representing a critical point where the degradation reaches its zenith. This information is crucial for practical applications, indicating that the ticarcillin antibiotic can be effectively utilized for up to 14 days before a noticeable decline in its concentration. Understanding the degradation kinetics of ticarcillin is essential for optimizing its use in various settings. Moreover, the inclusion of error bars in the graph enhances the reliability of our results by providing a visual representation of the standard errors associated with the mean concentrations.

**Figure 4:**
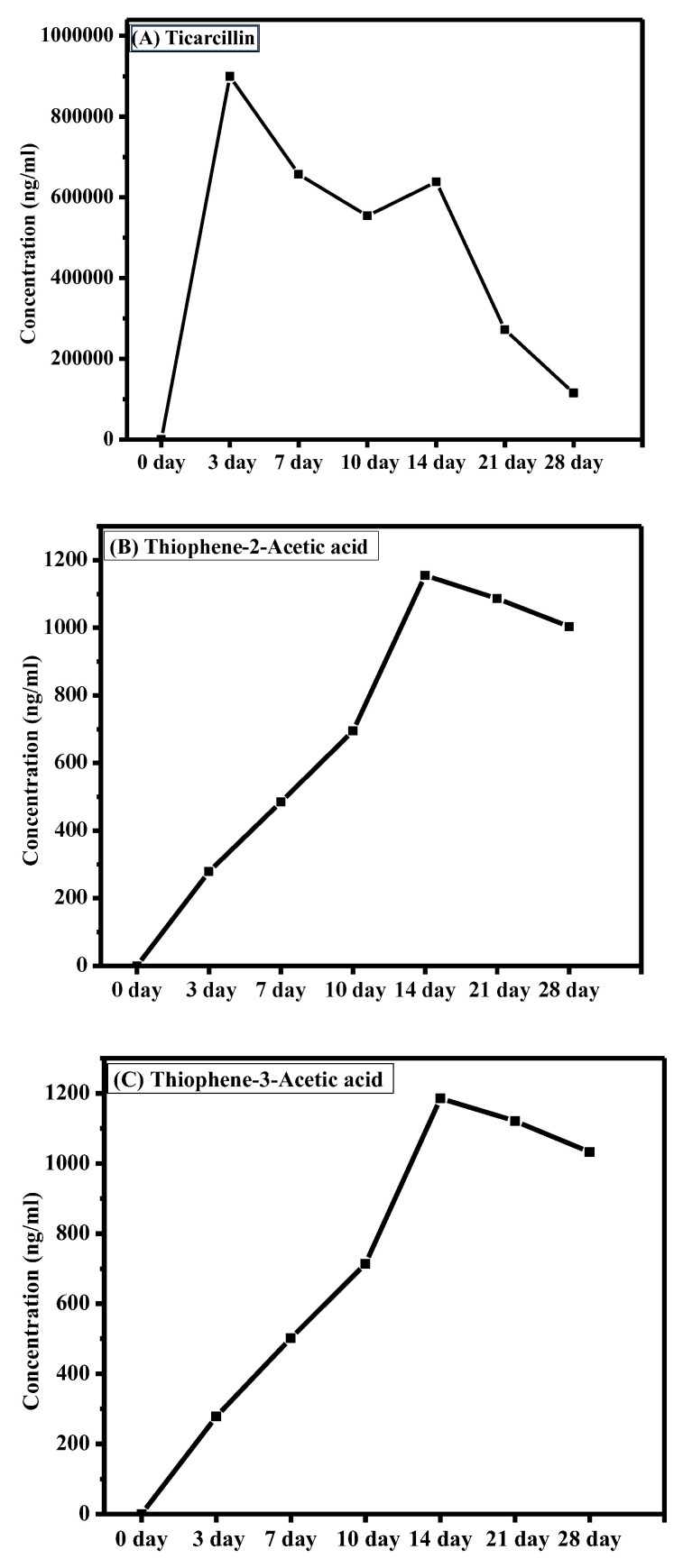
Changes in concentration of (**A**) timentin, (**B**) T2AA, and (**C**) T3AA over time.

The graph presented in Figure 4 illustrates the dynamic changes in ticarcillin and T2AA and T3AA levels over a 28-day period. The results reveal a discernible downtrend in the concentration of ticarcillin over the duration (see Table S3). This decline in concentration suggests its degradation into its metabolites. A notable increase in concentration of T2AA and T3AA is observed as shown in Figure 4. This elevation is particularly significant as ticarcillin is being degraded into T2AA and T3AA. The correlation between decreased concentration of ticarcillin and increased T2AA and T3AA levels suggests a potential link among these over the experimental period. This analytical approach not only enhances our understanding of antibiotic residues but also sets the stage for comprehensive investigations into the residue analysis of antibiotics in agricultural products. The presented methodology acts as a springboard for future explorations, encouraging further research in the realm of antibiotic residue analysis and monitoring in plant tissues.

### 2.4. Statistical Analysis

The particular characteristics of our data regarding timentin and its metabolites were taken into consideration when conducting an appropriate statistical analysis. ANOVA, or one-way analysis of variance, was used to compare the three groups. The statistical analysis revealed substantial differences (*p* < 0.0004) between the means of the three groups, indicating the significance of the findings of this study.

## 3. Materials and Methods

### 3.1. Standards and Reagents

Ticarcillin, Thiophene-2-Acetic acid, and Thiophene-3-Acetic acid were purchased from Sigma-Aldrich (St. Louis, MO, USA). LC-MS grade Acetonitrile (ACN) and Ammonium formate (LR grade) were obtained from Supelco (Merck, Darmstadt, Germany) and Fluka (Buchs, Switzerland). Ultrapure Milli-Q-Water was obtained from Milli-Q Academic apparatus (Millipore Corporation, Billerica, MA, USA) and was used for making solutions during analysis.

### 3.2. Plant Matrix and Sample Collection

Tomato cultivar Roma VF seeds were sterilized by treating them for a period of ten minutes with 15% sodium hypochlorite and then washing them for five times in sterile water. On half-strength Murashige and Skoog (MS) medium, the sterilized seeds were allowed to germinate in vitro. In all the trials, cotyledons from seedlings that were 7 days old before the first genuine leaves appeared were employed as beginning explants. Following the removal of the cotyledon base and tip, the explants were shifted such that the abaxial surface was in contact with the culture media. Explants under control were cultivated in MS media enriched with 1.0 mg/L BAP, 30 g/L sucrose, and 0.8% agar for gelling. The medium’s pH was set to 5.8 ± 0.2. The treatment explants were cultured on the same medium, additionally supplemented with a fresh 300 mg/L dose of timentin.

**Figure 5:**
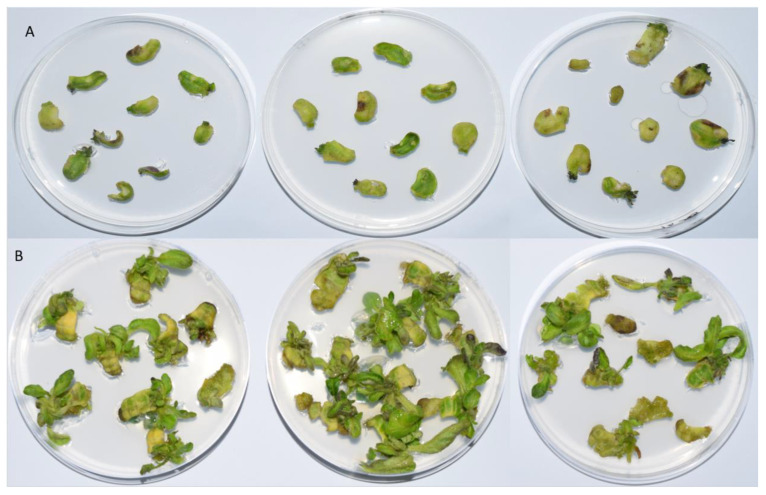
Explant growth after 28 days in basic media for cultivation enriched with (**A**) 1 mg/L BAP and (**B**) 1 mg/L BAP with 300 mg/L timentin. For each condition, three replicates are shown.

The plates were preserved at a temperature of 26/24 °C day/night, a relative humidity of 70%, and a light cycle of 16/8 h (Figure 5). On days 0, 14, and 28, the control samples were thawed in liquid nitrogen, while on days 3, 7, 10, 14, 21, and 28 the treatment samples were frozen. Prior to additional analysis, the samples were ground into a powder in liquid nitrogen. At least three repetitions of ten explants were used in each experiment.

### 3.3. Extraction Procedure

For quantitation of ticarcillin, Thiophene-2-Acetic acid, and Thiophene-3-Acetic acid, 0.500 g of sample was carefully weighed and placed into a 15 mL centrifuge tube, to which 0.500 mL of Milli-Q water was added and vortexed. Building upon this foundation, 5.00 mL of a 1% formic acid solution in methanol was introduced to the sample, which was then subjected once again to vertexing. Subsequent to this thorough mixing, the sample underwent centrifugation for ten minutes at 4 °C at 4500 revolutions per minute (rpm). This initial step aimed to ensure the even distribution of the sample within the solvent, setting the stage for subsequent precision.

Following centrifugation, the supernatant was filtered through a disposable membrane filter unit featuring a pore size of 0.45 µm. This filtration step is essential to eliminate any particulate matter that could interfere with subsequent analysis. Then, 1 mL of supernatant was diluted with 1 mL of mobile phase. The resultant extract was injected into LC-MS/MS for analysis.

### 3.4. Liquid Chromatography Tandem Mass Spectrometry (LC-MS/MS) Analysis

LC-MS/MS analysis was carried out using a SHIMADZU (Kyoto, Japan) model LC-30AD (Nexera X2) binary pump and LCMS-8060 Shimadzu spectrometer (Kyoto, Japan). Both the positive and negative Electrospray Ionization (ESI) modes were employed with the mass spectrometer. Nitrogen played a pivotal role in the process, serving as the heating and drying gas flow, ensuring precise and consistent conditions throughout the analysis. The mixture of 10 mM ammonium acetate and acetonitrile in a 10:90 ratio (*v*/*v*) was used as the mobile phase for analysis. The flow rate was adjusted at 0.300 mL/min. We employed a ZORBAX Eclipse Plus C18 column from Agilent Technologies, with dimensions of 4.6 mm in diameter and 100 mm in length, and a particle size of 3.5 µ. The volume of 10 µL was used for injection during analysis, and temperatures were carefully controlled at 5 °C and 30 °C for the auto sampler and column, respectively.

In terms of the MS conditions, both positive (+ve) and negative (−ve) ionization modes were employed for analysis, using ticarcillin, Thiophene-2-Acetic acid, and Thiophene-3-Acetic acid. The nebulizing gas flow rate was adjusted at 2.8 L/min, while the heating gas flow rate was 10 L/min. The interface temperature was maintained at 300 °C, the source temperature at 250 °C, and the heat block temperature at 400 °C. Finally, the drying gas flow rate was set at 10 L/min to ensure optimal performance of the LC-MS/MS method. The rinsing volume was set at 1000 µL, and the run time was 4.00 min. The retention times for ticarcillin, Thiophene-2-Acetic acid, and Thiophene-3-Acetic acid were determined to be 4.240, 3.815, and 3.804 min, respectively. The needle stroke was 48 mm, and rinsing was performed both before and after aspiration using the rinse port, for a duration of only 2 s.

In terms of MS conditions, ionization modes were +ve for ticarcillin and −ve for Thiophene-2-Acetic acid and Thiophene-3-Acetic acid. The dwell time was set at 100 ms, and Q1 Pre Bias (V) and Q3 Pre Bias (V) were adjusted for each analyte. Specifically, for ticarcillin, Q1 Pre Bias was −20.0 V and Q3 Pre Bias was −30.0 V, while for Thiophene-2-Acetic acid and Thiophene-3-Acetic acid, Q1 Pre Bias was 26.0 V and Q3 Pre Bias was 20.0 V, respectively. These comprehensive parameters collectively contribute to the robustness and reliability of the LC-MS/MS method, ensuring accurate and sensitive detection of ticarcillin and its degradation products in tomato leaves. The parameters of the experiment are also listed in Table S1.

### 3.5. Method Validation

The procedure for method validation adhered closely to the stringent guidelines established by the US Food and Drug Administration (FDA). This rigorous validation aimed to thoroughly evaluate the precision, accuracy, specificity, and linearity of the method used for quantifying ticarcillin in tomato leaves. Optimized Multiple Reaction Monitoring (MRM) parameters were employed for the analysis of ticarcillin and its degradation products, specifically Thiophene-2-Acetic acid and Thiophene-3-Acetic acid (Table 1). The MRM mode was employed to track the protonated molecules ([M+H]^+^), precursor ions, and diagnostic product ions. By directly infusing individual solutions and a combination of standard chemicals at a concentration of 0.1 μg/mL, the MRM parameters were optimized for every analyte. Three periods were used in the MRM sequence, which was conducted in order to monitor various transition pairs with parameters that were optimized for each period. Ticarcillin’s protonated molecule was observed at *m*/*z* 385.15, with three corresponding product ions at *m*/*z* 160.05, 114.05, and 225.95. The dwell time for each transition was set at 100 milliseconds, allowing for precise data acquisition. Collision energy settings were optimized to achieve optimal fragmentation, and pre-bias voltages were applied for stable ionization and transmission. The ionization mode for ticarcillin was positive. For Thiophene-2-Acetic acid, the precursor ion was observed at *m*/*z* 141.25, with three corresponding product ions at *m*/*z* 45.00, 97.05, and 77.00. Similar to ticarcillin, a dwell time of 100 milliseconds was applied for each transition, and collision energy settings (24, 11, and 39 eV) were optimized. The negative ionization mode was used for Thiophene-2-Acetic acid. Moreover, Thiophene-3-Acetic acid exhibited a precursor ion at *m*/*z* 141.15, with product ions at *m*/*z* 97.15, 45.05, and 77.10. The MRM transitions were monitored with a dwell time of 100 milliseconds, and collision energy settings (11, 23, and 34 eV) were tailored for each transition. The negative ionization mode was also applied for Thiophene-3-Acetic acid. The conditions specified in Table 1 were followed to obtain the most abundant MRM transitions for each analyte. The table provides information on the collision energies, precursor ions, and product ions for ticarcillin, Thiophene-2-Acetic acid, and Thiophene-3-Acetic acid.

As part of this evaluation, three distinct quality control limits, referred to as QCs, were established at varying concentration levels. These control limits included a high-quality control limit (QCH) set at 2500 ng/mL, a medium-quality control limit (QCM) at 1000 ng/mL, and a low-quality control limit (QCL) at 500 ng/mL. The primary objective behind these quality controls was to assess the linearity, precision (both intra-day and inter-day), and overall accuracy of the ticarcillin assay. In each validation run, six control samples were tested at each concentration level (QCH, QCM, QCL), alongside a calibration curve. To accurately quantify small molecules in tomato leaves, representative chromatograms of ticarcillin, Thiophene-2-Acetic acid, and Thiophene-3-Acetic acid were spiked and obtained using retention time and multiple reaction monitoring (MRM). The precision and accuracy of the developed LC-MS/MS method were thoroughly assessed through intra-day and inter-day evaluations, employing quality control samples at low (LQC), medium (MQC), and high (HQC) concentration levels. The data presented in Table 2 summarize these assessments, with each compound’s concentration (ng/mL) and corresponding accuracy and precision values outlined. This information was derived from the analysis of quality control samples, with six replicates for each at the low, medium, and high concentration levels. The precision, expressed as a percentage coefficient of variation (% CV), was consistently within a ±15% range, while the accuracy remained within the ±15% margin. By combining the outcomes of six individual assay replicates from quality controls across three distinct batch runs carried out on two distinct days, the accuracy for inter-run and intra-run evaluations was ascertained. The accuracy of the assay for QC values was calculated using the equation ((mean value/nominal value) × 100), and the precision (% CV) was calculated using the equation ((standard deviation/mean) × 100). The inter-run and intra-run precision (% CV) consistently fell within ±15%, and inter-run and intra-run accuracy ranged between 85% and 115%. All the data presented in Table 2 unequivocally demonstrate the precision and accuracy of the method. The R^2^ value represents the linear regression, with all results reported in units of ng/mL. The lowest concentration of an analyte that can be reliably detected refers to lower limit of detection (LOD). It was determined using the Signal-to-Noise approach, reducing analyte concentrations until a response equivalent to three times the detected background level was achieved. This approach adheres to the generally accepted detection limit for the signal-to-noise ratio of “three”.

## 4. Conclusions

In summary, the current study aimed to develop a novel LC-MS/MS-based method for accurately measuring the degradation products of ticarcillin in the tomato leaf. The findings indicate that the LC-MS/MS method used was suitable and reliable for quantifying ticarcillin, Thiophene-2-Acetic acid, and Thiophene-3-Acetic acid. In this study, we conducted a comprehensive analysis of ticarcillin and its degradation products, Thiophene-2-Acetic acid and Thiophene-3-Acetic acid, in tomato leaves using LC-MS/MS. Our validation process adhered to stringent guidelines established by the US Food and Drug Administration (FDA), ensuring the precision, accuracy, specificity, and linearity of the quantification method. The results demonstrated a robust linear range of 500.0 to 3500.0 ng/mL, with outstanding regression (R^2^ = 0.99). Representative chromatograms confirmed the presence of ticarcillin and its metabolites, showcasing distinct peaks at their specific retention times. Sample analysis revealed the degradation of timentin into its metabolites over time, with the highest concentration observed on the 14th day. This information is valuable for determining the shelf life and effective usage period of timentin.

Overall, this research provides a reliable and validated method for the quantification of ticarcillin and its degradation products in tomato leaves, contributing to the understanding of antibiotic degradation in plant matrices. This analytical approach can be instrumental in assessing antibiotic residue levels in agricultural products and ensuring food safety and security.

## Figures and Tables

**Table 1:** MRM transitions of ticarcillin and its degradation products.

Compounds	Precursor (Q1) (*m*/*z*)	Product (Q3) (*m*/*z*)	Dwell Time (ms)	CE (eV)	Q1 Pre Bias (V)	Q3 Pre Bias (V)	Ionization
Ticarcillin	385.15	160.05	100	−14	−20	−30	Positive
	385.15	114.05	100	−36	−20	−20	
	385.15	225.95	100	−13	−20	−22	
Thiophene-2- Acetic acid	141.25	45.00	100	24	14	11	Negative
	141.25	97.05	100	11	14	25	
	141.25	77.00	100	39	12	20	
Thiophene-3-Acetic acid	141.15	97.15	100	11	26	20	Negative
	141.15	45.05	100	23	14	12	
	141.15	77.10	100	34	13	11	

**Table 2:** Intra-day and inter-day precision and accuracy.

S.No	Compound Name	Quality Control	Concentration (ng/mL)	Intra-day		Inter-day		R^2^	LOD (ng/mL)	LOQ (ng/mL)	Linearity Range (ng/mL)
				Accuracy %	Precision (% CV)	Accuracy %	Precision (% CV)				
1	Ticarcillin	LQC	500	110.1	3.2	107.00	2.9	0.9957	59.0	178.8	500 to 3500
		MQC	1000	110.9	1.9	109.5	2.2				
		HQC	2500	104.4	1.3	103.6	1.2				
2	Thiophene-2- Acetic acid	LQC	500	99.7	9.1	98.6	7.4	0.9990	120.3	364.7	500 to 3500
		MQC	1000	105.8	1.9	103.9	2.2				
		HQC	2500	101.7	2.1	99.7	1.8				
3	Thiophene-3-Acetic acid	LQC	500	102.9	6.6	100.7	6.5	0.9982	156.2	473.6	500 to 3500
		MQC	1000	108.0	2.7	105.5	3.1				
		HQC	2500	104.5	1.7	101.7	1.9				

CV: Coefficient of variation.

## Data Availability

The raw data is available upon request.

## Supplementary Materials

The supplementary material can be found online at https://www.mdpi.com/article/10.3390/antibiotics13020133/s1, Figure S1: Calibration curve of (A) Ticarcillin (B) Thiophene-2-Acetic acid (C) Thiophene-3-Acetic acid to determine the quality of the samples; Table S1: The other operating parameters of the LC-MS/MS method; Table S2: Abundance of Ticarcillin and its degradation products; Table S3: The degradation data of Ticarcillin and TAA 2&3 over period of time.

## Abbreviations

TAA	Thiophene acetic acid
T2AA	Thiophene-2-Acetic acid
T3AA	Thiophene-3-Acetic acid
ACN	Acetonitrile
µL	Microliter
L	Liter
°C	Degree Celsius
+ve	Positive
−ve	Negative
Min	Minute
mM	Milli molar
gm	Gram
*v*/*v*	volume/volume

## References

[1] Dias J.P.T. (2019). Plant Growth Regulators in Horticulture: Practices and Perspectives. Biotecnol. Veg..

[2] Sabagh A.E., Mbarki S., Hossain A., Iqbal M.A., Islam M.S., Raza A., Llanes A., Reginato M., Rahman M.A., Mahboob W. (2021). Potential Role of Plant Growth Regulators in Administering Crucial Processes against Abiotic Stresses. Front. Agron..

[3] Zhao B., Liu Q., Wang B., Yuan F. (2021). Roles of Phytohormones and Their Signaling Pathways in Leaf Development and Stress Responses. J. Agric. Food Chem..

[4] Gill K., Kumar P., Negi S., Sharma R., Joshi A.K., Suprun I.I., Al-Nakib E.A. (2023). Physiological Perspective of Plant Growth Regulators in Flowering, Fruit Setting and Ripening Process in Citrus. Sci. Hortic..

[5] Ali S., Baloch A.M. (2020). Overview of Sustainable Plant Growth and Differentiation and the Role of Hormones in Controlling Growth and Development of Plants under Various Stresses. Recent Pat. Food Nutr. Agric..

[6] Mok M.C. (2019). Cytokinins and Plant Development—An Overview. Cytokinins.

[7] Sabagh A.E., Hossain A., Islam M.S., Iqbal M.A., Amanet K., Mubeen M., Nasim W., Wasaya A., Llanes A., Ratnasekera D. (2021). Prospective Role of Plant Growth Regulators for Tolerance to Abiotic Stresses. Plant Growth Regulators.

[8] Shah A., Nazari M., Antar M., Msimbira L.A., Naamala J., Lyu D., Rabileh M., Zajonc J., Smith D.L. (2021). PGPR in Agriculture: A Sustainable Approach to Increasing Climate Change Resilience. Front. Sustain. Food Syst..

[9] Wu X., Gong D., Zhao K., Chen D., Dong Y., Gao Y., Wang Q., Hao G. (2023). Research and Development Trends in Plant Growth Regulators. Adv. Agrochem.

[10] Vergara R., Olivares F., Olmedo B., Toro C., Muñoz M., Zúñiga C., Mora R., Plantat P., Miccono M., Loyola R. (2021). Gene Editing in *Prunus* spp.: The Challenge of Adapting Regular Gene Transfer Procedures for Precision Breeding. Prunus—Recent Advances.

[11] Heap B., McAinsh M., Toledo-Ortiz G. (2022). RootTarget: A Dynamic Model Enabling the Targeted Application of Plant Growth Regulators for Rice. Plants People Planet.

[12] Li S.-B., Xie Z.-Z., Hu C.-G., Zhang J.-Z. (2016). A Review of Auxin Response Factors (ARFs) in Plants. Front. Plant Sci..

[13] Emenecker R.J., Strader L.C. (2020). Auxin-Abscisic Acid Interactions in Plant Growth and Development. Biomolecules.

[14] Enders T.A., Strader L.C. (2015). Auxin Activity: Past, Present, and Future. Am. J. Bot..

[15] Sanchez-Corrionero A., Sánchez-Vicente I., Arteaga N., Manrique-Gil I., Gómez-Jiménez S., Torres-Quezada I., Albertos P., Lorenzo O. (2023). Fine-Tuned Nitric Oxide and Hormone Interface in Plant Root Development and Regeneration. J. Exp. Bot..

[16] Guan L., Tayengwa R., Cheng Z., Peer W.A., Murphy A.S., Zhao M. (2019). Auxin Regulates Adventitious Root Formation in Tomato Cuttings. BMC Plant Biol..

[17] Gonin M., Bergougnoux V., Nguyen T.D., Gantet P., Champion A. (2019). What Makes Adventitious Roots?. Plants.

[18] Schneider A., Godin C., Boudon F., Demotes-Mainard S., Sakr S., Bertheloot J. (2019). Light Regulation of Axillary Bud Outgrowth along Plant Axes: An Overview of the Roles of Sugars and Hormones. Front. Plant Sci..

[19] Jamil M., Saher A., Javed S., Farooq Q., Shakir M., Zafar T., Komal L., Hussain K., Shabir A., Javed A. (2021). A Review on Potential Role of Auxins in Plants, Current Applications and Future Directions. J. Biodivers. Environ. Sci..

[20] Fenn M.A., Giovannoni J.J. (2021). Phytohormones in Fruit Development and Maturation. Plant J..

[21] Shi Q., Zhang Y., To V.-T., Shi J., Zhang D., Cai W. (2020). Genome-Wide Characterization and Expression Analyses of the Auxin/Indole-3-Acetic Acid (Aux/IAA) Gene Family in Barley (*Hordeum vulgare* L.). Sci Rep.

[22] Grossmann K. (2010). Auxin Herbicides: Current Status of Mechanism and Mode of Action. Pest Manag. Sci..

[23] Costa M.G.C., Nogueira F.T.S., Figueira M.L., Otoni W.C., Brommonschenkel S.H., Cecon P.R. (2000). Influence of the Antibiotic Timentin on Plant Regeneration of Tomato (*Lycopersicon esculentum* Mill.) Cultivars. Plant Cell Rep..

[24] George S., Rafi M., Aldarmaki M., ElSiddig M., Nuaimi M.A., Sudalaimuthuasari N., Nath V.S., Mishra A.K., Hazzouri K.M., Shah I. (2023). Ticarcillin Degradation Product Thiophene Acetic Acid Is a Novel Auxin Analog That Promotes Organogenesis in Tomato. Front. Plant Sci..

[25] Alfei S., Schito A.M. (2022). β-Lactam Antibiotics and β-Lactamase Enzymes Inhibitors, Part 2: Our Limited Resources. Pharmaceuticals.

[26] Varlamova N.V., Dolgikh Y.I., Blinkov A.O., Baranova E.N., Khaliluev M.R. (2021). Effects of Different β-Lactam Antibiotics on Indirect Tomato (*Solanum lycopersicum* L.) Shoot Organogenesis and Agrobacterium Tumefaciens Growth Inhibition In Vitro. Antibiotics.

[27] Ling H.-Q., Kriseleit D., Ganal M.W. (1998). Effect of Ticarcillin/Potassium Clavulanate on Callus Growth and Shoot Regeneration in Agrobacterium-Mediated Transformation of Tomato (*Lycopersicon esculentum* Mill.). Plant Cell Rep..

[28] Li Z., Liu G., Fang F., Bao M. (2007). Adventitious Shoot Regeneration of Platanus Acerifolia Willd. Facilitated by Timentin, an Antibiotic for Suppression of Agrobacterium Tumefaciens in Genetic Transformation. For. Stud. China.

[29] Estaji A., Chamani E., Khazaei Z. (2021). Influence of Plant Growth Regulators on Callogenesis and the Biomass of Cell Suspensions in Lily (*Lilium ledebourii* and *Lilium regal*). J. Appl. Biotechnol. Rep..

[30] Hazubska-Przybył T., Ratajczak E., Obarska A., Pers-Kamczyc E. (2020). Different Roles of Auxins in Somatic Embryogenesis Efficiency in Two Picea Species. Int. J. Mol. Sci..

[31] Grzebelus E., Skop L. (2014). Effect of β-Lactam Antibiotics on Plant Regeneration in Carrot Protoplast Cultures. Vitr. Cell. Dev. Biol.-Plant.

[32] Gudiño M.E., Blanco-Touriñán N., Arbona V., Gómez-Cadenas A., Blázquez M.A., Navarro-García F. (2018). β-Lactam Antibiotics Modify Root Architecture and Indole Glucosinolate Metabolism in Arabidopsis Thaliana. Plant Cell Physiol..

[33] IJMS|Free Full-Text|Integrating the Roles for Cytokinin and Auxin in De Novo Shoot Organogenesis: From Hormone Uptake to Signaling Outputs. https://www.mdpi.com/1422-0067/22/16/8554.

[34] Cao Z., Duan X., Yao P., Cui W., Cheng D., Zhang J., Jin Q., Chen J., Dai T., Shen W. (2017). Hydrogen Gas Is Involved in Auxin-Induced Lateral Root Formation by Modulating Nitric Oxide Synthesis. Int. J. Mol. Sci..

