# Supplementary material for: Advancing Antibiotic Residue Analysis: LC-MS/MS Methodology for Ticarcillin Degradation Products in Tomato Leaves
Source: Antibiotics (Basel). 2024 Jan 29;13(2):133. doi: 10.3390/antibiotics13020133 (PMC10886401; doi:10.3390/antibiotics13020133)
Supplement: Supplementary file 1 [file antibiotics-13-00133-s001.zip › antibiotics-2778219-supplementary.pdf]

Supplementary information

Article

Advancing Antibiotic Residue Analysis: LC-MS/MS Methodology for Ticarcillin Degradation Products in Tomato Leaves

Table S1. The other operating parameters of the LC-MS/MS method.

LC parameters	Rinsing volume	1000 µL
	Run time	4.00 min
	Retention time	Ticarcillin = 4.240 min<br>Thiophene-2-Acetic acid = 3.815 min<br>Thiophene-3-Acetic acid = 3.804 min
	Needle Stroke	48 mm
	Rinse Mode	Before and after aspiration
	Rinse Method	Rinse port only
	Rinse Time	2 sec
MS conditions	Mode of ionization	+ve (Ticarcillin), -ve (TAA)
	Dwell time	100 msec
	Q1 Pre Bias (V)	−20.0 (Ticarcillin), 26.0 (TAA)
	Q3 Pre Bias (V)	−30.0 (Ticarcillin), 20.0 (TAA)

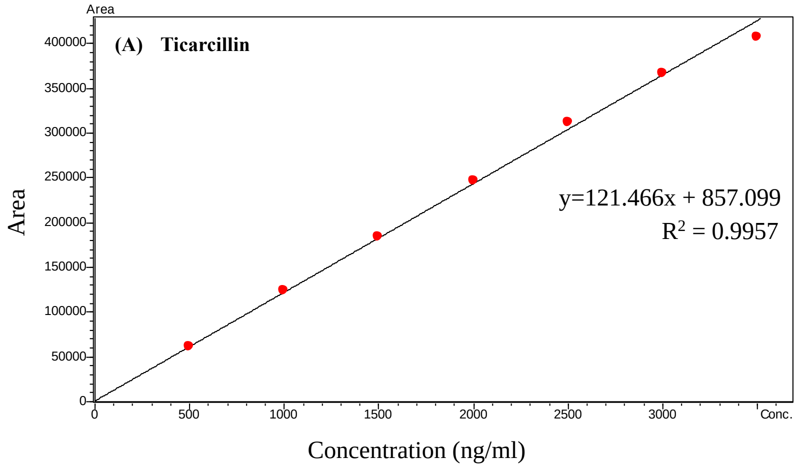

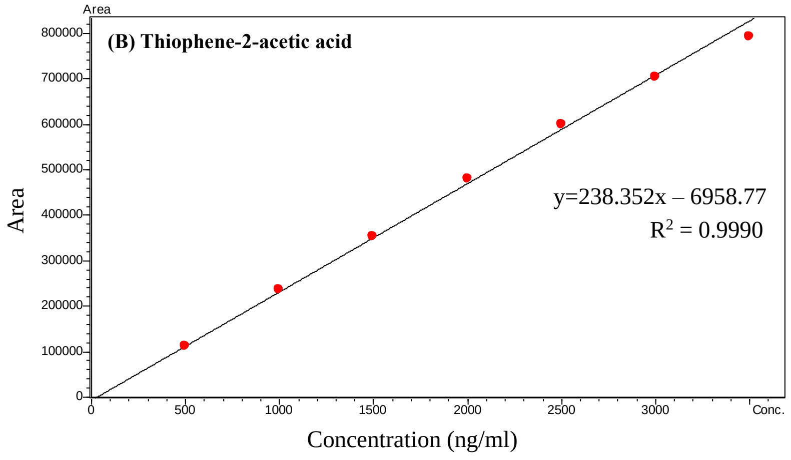

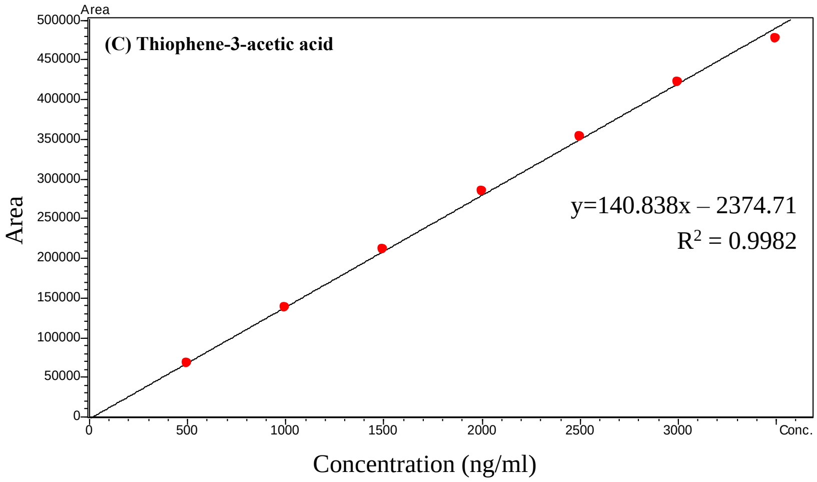

Figure S1. Calibration curve of (**A**) Ticarcillin (**B**) Thiophene-2-Acetic acid (**C**) Thiophene-3-Acetic acid to determine the quality of the samples.

Table S2. Abundance of Ticarcillin and its degradation products.

S.No	Sample Name	Area (Abundance)
1	Ticarcillin	78714372
2	Thiophene-2 acetic acid	270966
3	Thiophene-3-acetic acid	169201

Table S3. The degradation data of Ticarcillin and TAA 2&3 over period of time.

	Concentration (ng/ml)		
**Sample**	**Ticarcillin**	**Thiophene-2 Acetic Acid**	**Thiophene-3 Acetic Acid**
0 day	0	0	0
3 day	899548.344	278.772	278.113
7 day	657518.807	484.316	501.761
10 day	553862.969	694.355	713.516
14 day	638211.272	1154.517	1185.493
21 day	271747.321	1086.739	1120.841
28 day	115286.508	1003.427	1032.241
